# Comparison of the effect of argon, hydrogen, and nitrogen gases on the reduced graphene oxide-hydroxyapatite nanocomposites characteristics

**DOI:** 10.1186/s13065-020-00712-3

**Published:** 2020-10-07

**Authors:** Hassan Nosrati, Rasoul Sarraf-Mamoory, Arman Karimi Behnagh, Reza Zolfaghari Emameh, Amir Aidun, Dang Quang Svend Le, Maria Canillas Perez, Cody Eric Bünger

**Affiliations:** 1 Department of Materials Engineering, Tarbiat Modares University, Tehran, Iran; 2 Faculty of Medicine, Iran University of Medical Science, Tehran, Iran; 3 Department of Energy and Environmental Biotechnology, National Institute of Genetic Engineering and Biotechnology (NIGEB), 14965/161, Tehran, Iran; 4 National Cell Bank of Iran, Pasteur Institute of Iran, Tehran, Iran; 5 Tissues and Biomaterials Research Group (TBRG), Universal Scientific Education and Research Network (USERN), Tehran, Iran; 6 Department of Clinical Medicine, Aarhus University, Aarhus, Denmark; 7 Instituto de Cerámica Y Vidrio, CSIC, Madrid, Spain

**Keywords:** Argon, Hydrogen, Nitrogen, Graphene, Hydroxyapatite, Nanocomposite

## Abstract

In this study, the effect of the argon, nitrogen, and hydrogen gases on the final properties of the reduced graphene oxide- hydroxyapatite nanocomposites synthesized by gas injected hydrothermal method was investigated. Four samples were synthesized, which in the first sample the pressure was controlled by volume change at a constant concentration. In subsequent samples, the pressure inside the autoclave was adjusted by the injecting gases. The initial pressure of the injected gases was 10 bar and the final pressure considered was 25 bar. The synthesized powders were consolidated at 950 °C and 2 MPa by spark plasma sintering method. The final samples were subjected to Vickers indentation analysis. The findings of this study indicate that the injection of argon, hydrogen, and nitrogen gases improved the mechanical properties of the nanocomposites. Injection of gases increased the crystallinity and particle size of hydroxyapatite, and this increase was greater for nitrogen gas than for others. Injection of these gases increased the rate of graphene oxide reduction and in this case the effect of nitrogen gas was greater than the others. 
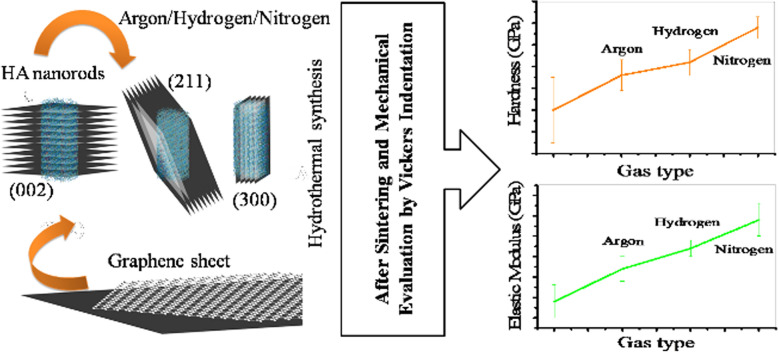

## Introduction

Calcium phosphates have been widely used in the medical field. Members of this family include hydroxyapatite (HA), Tetracalcium phosphate (TeCP), α- Tricalcium phosphate (α-TCP), β- Tricalcium phosphate (β-TCP), dicalcium phosphate dehydrate (DCPD), dicalcium phosphate anhydrous (DCPA), and octacalcium phosphate (OCP). Among these bioceramics, HA is less soluble in the biological environment and therefore suitable for orthopedic applications as an implant [1, 2]. HA is synthesized in a variety of ways, including combustion preparation, solid-state reaction, electrochemical deposition, sol–gel, hydrolysis, precipitation, sputtering, multiple emulsion, biomimetic deposition, solvothermal method, and hydrothermal process [3–15]. The variety of methods has made it possible to synthesize these ceramics in various forms such as rods, wires, ribbons, and tubes [16–21]. HA (Ca_10_(PO_4_)_6_(OH)_2_) has unique biomaterial properties such as resemblance to bone mineral, biocompatibility, bioactivity, osteoconductivity, non-immunogenicity, and non-toxicity. But still, it has poor mechanical properties such as intrinsic brittleness, low fracture toughness (fracture toughness of 0.28–1.08 MPa.m^0.5^), poor tensile strength, and weak wear resistance which have limited the scope of its applications [22–31]. Researchers have tried various ways to overcome this weakness, including reducing particle size using nanotechnology techniques and adding reinforcing materials. Graphene and its derivatives have been widely used because of their excellent mechanical properties and high specific surface area among the reinforcing materials used so far. Graphene is a member of the carbon nanomaterial family that has a honeycomb structure with a carbon atom thick. It is biocompatible and has been widely used in medical sciences including drug delivery, orthopedics, and bioimaging [32–49].

Different methods have been applied to synthesize hydroxyapatite and graphene nanocomposites so far. Among these methods, the hydrothermal process has received much attention because of its lack of calcination stage, excellent control and the ability to synthesize in situ. In this method, precursors containing calcium and phosphate ions are used as suppliers of the elements required for hydroxyapatite and graphene oxide as the graphene precursor. The high-pressure hydrothermal process results in the synthesis of hydroxyapatite and the reduction of graphene oxide (The more the graphene oxide sheets reduce the better mechanical properties of the synthesized composites). During the process, calcium ions first bind to the graphene oxide surface agents by ionic bond, and finally, in reaction with phosphate ions, hydroxyapatite is synthesized in situ on the surface of the graphene sheets. The synthesized powders are consolidated in various ways such as spark plasma sintering (SPS) and hot pressing (HP). Recently, gas injection into hydrothermal autoclave has been used to increase the rate of graphene oxide reduction. Increasing hydrothermal pressure by injecting gas also improves the mechanical properties of hydroxyapatite. Increasing the hydrothermal pressure in this method increases the crystallinity and crystallite size of HA. But the type of gas injected will affect the final mechanical properties obtained. Previously, researchers have shown that nitrogen gas reduces graphene oxide more than hydrogen gas [50–63].

To investigate the effect of the type of injected gas on the final mechanical properties, three types of gas including argon, nitrogen, and hydrogen are considered in this study and the pressure of the gases was assumed to be constant. The synthesized powders were then consolidated by SPS and examined for mechanical properties. Characterization methods for synthesized powders and sintered samples include X-ray diffraction (XRD), Raman spectroscopy, Fourier transform infrared spectroscopy (FTIR), field emission scanning electron microscopy (FESEM), transmission electron microscopy (TEM), high angle annular dark field (HAADF), energy-dispersive X-ray spectroscopy (EDS) and Vickers indentation technique. The results obtained in this study are expected to be very useful for similar researches.

## Experimental

**Figure 1:**
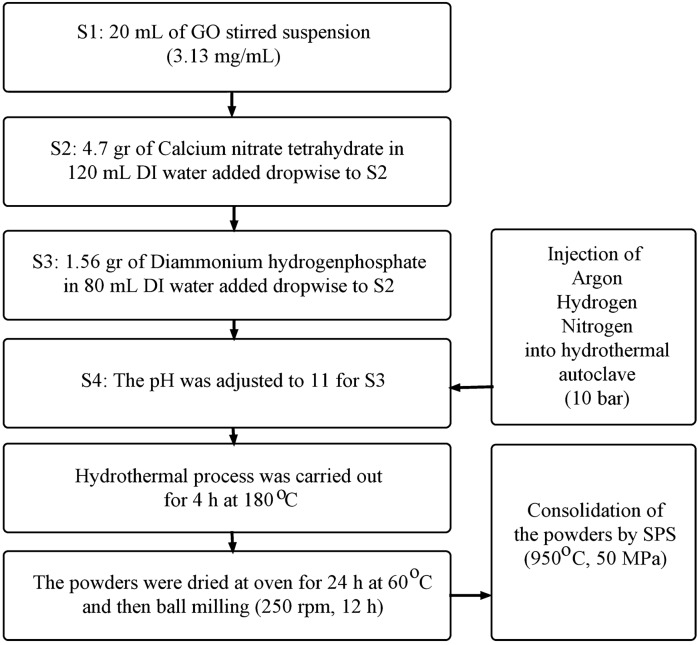
The steps for preparing powders and sintered samples

The chemicals used in this study include calcium nitrate tetrahydrate (Merck, > 99%), diammonium hydrogenphosphate (Merck, > 99%), ammonium solution (Merck, 25%), and GO (Abalonyx, 25 g/L DMF). Figure 1 shows the steps for preparing powdered and sintered samples. In this chart, solution 1 (S1) was stirred for 1 h and pH was adjusted with ammonium solution. Finally, four samples were synthesized and the pressure applied was equal in all samples (I, II, III, IV). In sample I, the volume of the solution was changed by keeping the concentration constant to reach the desired pressure. Table 1 shows the specifications for the samples. In these samples the final pressure was assumed to be 25 bar, and in all of them the graphene content was considered to be 1.5 wt%.

**Table 1:** Specifications for the samples

Samples	Specification
I	Using volume change to set the pressure
II	10 bar Argon gas injection
III	10 bar Hydrogen gas injection
IV	10 bar Nitrogen gas injection

For sintering of the powders, SPS method was used (Dr Sinter SPS-1050-CE). Details of the sintering operation have already been described in detail [64, 65].

### Characterization

Table 2 shows the specifications of the devices and the methods used for characterization of powders and consolidated samples. Instrumented microindentation experiments were conducted on the polished surfaces of samples using Grindosonic tester with a Vickers tip at a maximum load of 1 N (ramp dwell time of 10 s). Seven tests were performed at different locations of each sample. Elastic modulus and hardness were calculated from the load–displacement curve using Oliver-Pharr method [66]. The Archimedes method was used to calculate the relative density of sintered samples (ASTM C373-88).

**Table 2:** Specifications of the devices and the methods used for characterization of powders and consolidated samples

Method	Device	Specification
XRD	X’ Pert Pro, Panalytical Co	Cu Kα radiation (λ = 1.5406 Å, 40 kV, 40 mA)
Raman spectroscopy	Renishaw inVia spectrometer	Wavelength of 532 nm, green laser (recording 5 times for 10 s of each accumulation)
FTIR	VERTEX 70, Bruker Corp	Applying 200 MPa pressures (1 mm thickness)
FESEM	Hitachi S4700 equipped with energy dispersive X-ray spectroscopy	Au coated by sputtering
TEM	CM120, Philips	–
HAADF, EDS	TALOS F200A with a twin lens system	using TALOS microscope in STEM mode, exposure times of 5 min were used to create elemental distribution maps
ICP	DV7300, Optima Co	–

Eq. 1 was used to evaluate the crystallinity of HA (Xc) [67].

1 $${\text{X}}_{{\text{c}}} = { 1} - \frac{{{\upnu }_{{\left( {112/{ }300} \right)}} }}{{{\text{I}}_{300} }}$$

where υ_(112/300)_ and I_300_ are the intensity of the hollow between diffraction peaks of HA in the planes (300) and (112) and the intensity of the peak of HA in the plane (300), respectively. Eq. 2 was used to evaluate the crystallite size (Williamson-Hall method) [68].

2 $$\beta .{\text{Cos }}\theta = \frac{{\left( {0.9{\uplambda }} \right)}}{{\text{d}}} + { 4}\varepsilon .{\text{Sin }}\theta$$

In this equation, d, θ, and λ are grain size, Bragg diffraction angle, and wave length of used X-ray (Cu), respectively. β and ε are full width at half height (FWHM), and crystalline lattice strain, respectively. Diamond 3.2, ImageJ, and Origin pro 2016 were used in this study for drawing shapes and graphs.

## Results and discussion

**Fig. 2:**
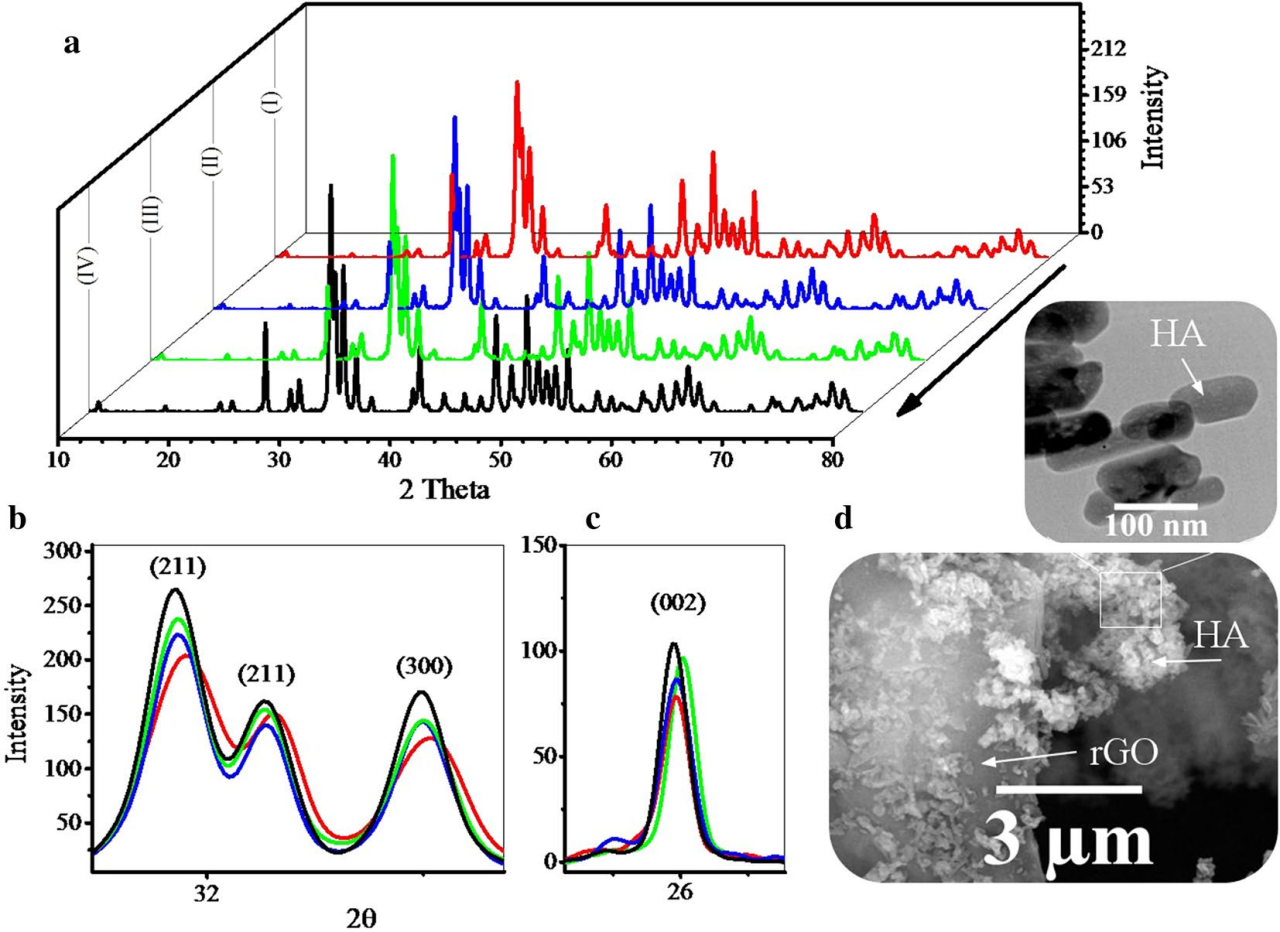
**a**–**c** XRD patterns of the synthesized powders using volume change (I), argon (II), hydrogen (III), and nitrogen (IV) gas injection (10 bar) along with the **d** FESEM and the TEM images of the IV powders

Figure 2 shows the XRD pattern of the synthesized powders along with the FESEM and the TEM images for IV powders. The patterns obtained for all powders are consistent with the XRD pattern of pure HA (Fig. 2a). Thus, the HA synthesized in these powders is pure and has a hexagonal structure. The main directions of growth in HA crystals are (211), (300), and (002). A closer look at these patterns reveals that gas injection has increased the peaks intensity (Fig. 2b, c) [52, 64, 65]. But this increase is greater for nitrogen gas than others. Probably, because of the lower solubility of nitrogen gas in water, the pressure drop at higher temperatures is lower for this gas and causes higher pressure. The crystallinity and crystallite size of the sample synthesized with nitrogen gas are also slightly higher than the others. As XRD patterns show, there is no sign of the presence of graphene sheets. But in the FESEM image (Fig. 2d), the presence of graphene sheets is clearly evident. Graphene oxide used as a precursor has a peak at 2theta≈10. As a result of the reduction of GO, this peak is removed and a short, wide peak appears at 2theta≈26. The absence of peaks at 2theta≈10 indicates that the graphene oxide was reduced in all samples. Reduced graphene oxide has an amorphous structure and its broad peak is covered with (002) planes of HA at 2theta≈26. In a previous report, pure GO was exposed to similar hydrothermal conditions, and the results of XPS confirmed that it was well reduced [24]. The TEM image of the HA particles shows that the morphology of these particles is nanorods with diameters of about 40 nm and longitudinally variable. In a similar study, it was found that the growth of these nanorods was (002) planes (c axis) [63, 69].

**Scheme 1:**
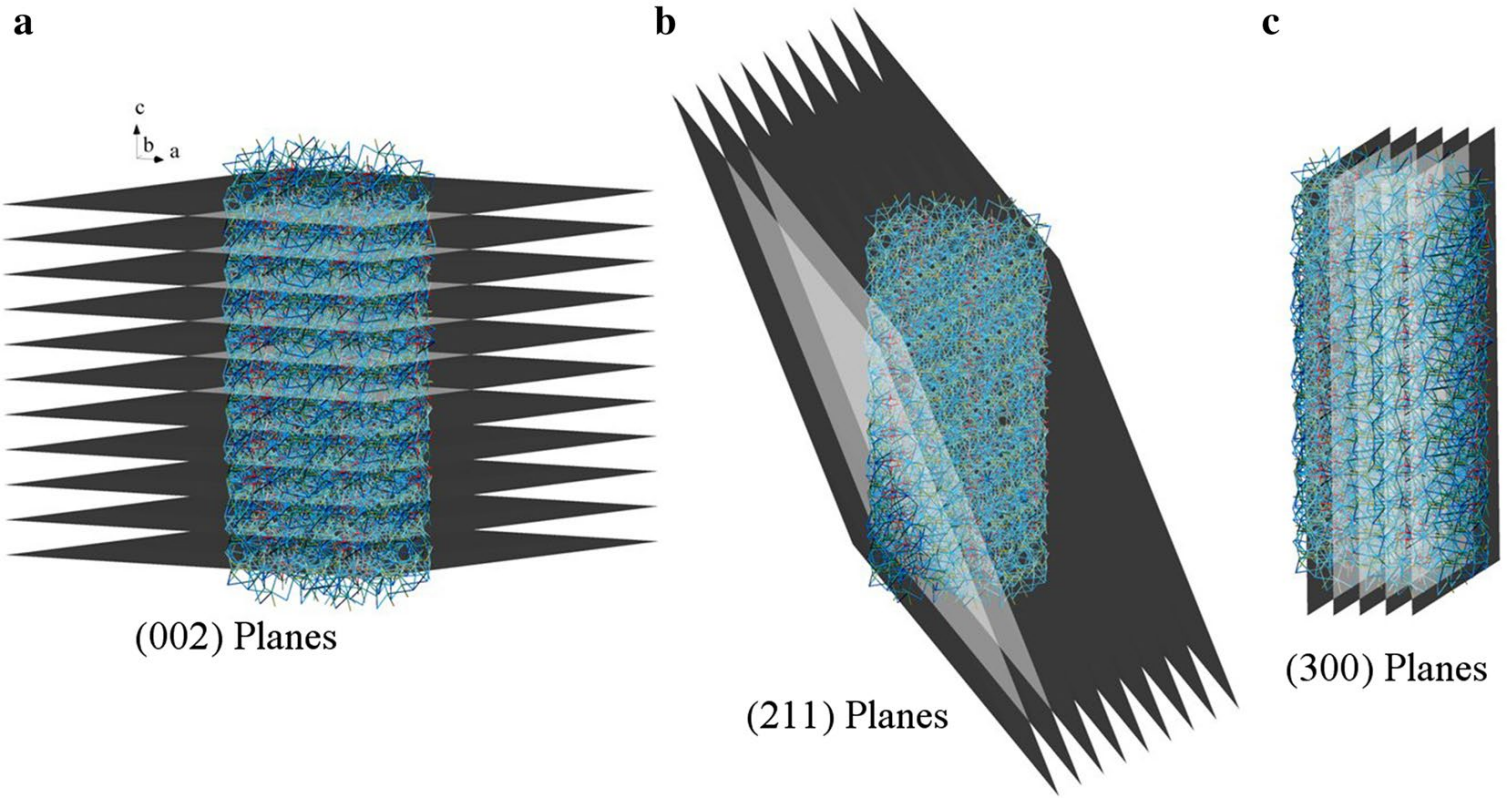
**a** (002) planes, **b** (211) planes, and **c** (300) planes of HA, which play a major role in the growth of crystals (the main crystalline planes of HA)

Scheme 1 illustrates the main crystalline planes of HA, which play a major role in the growth of crystals. As mentioned earlier, (002) planes have a more dominant role in this study than other planes (c axis). (002) planes are perpendicular to (300) planes, and the angle between (300) planes and (211) planes is about 27 degrees [65].

**Fig. 3:**
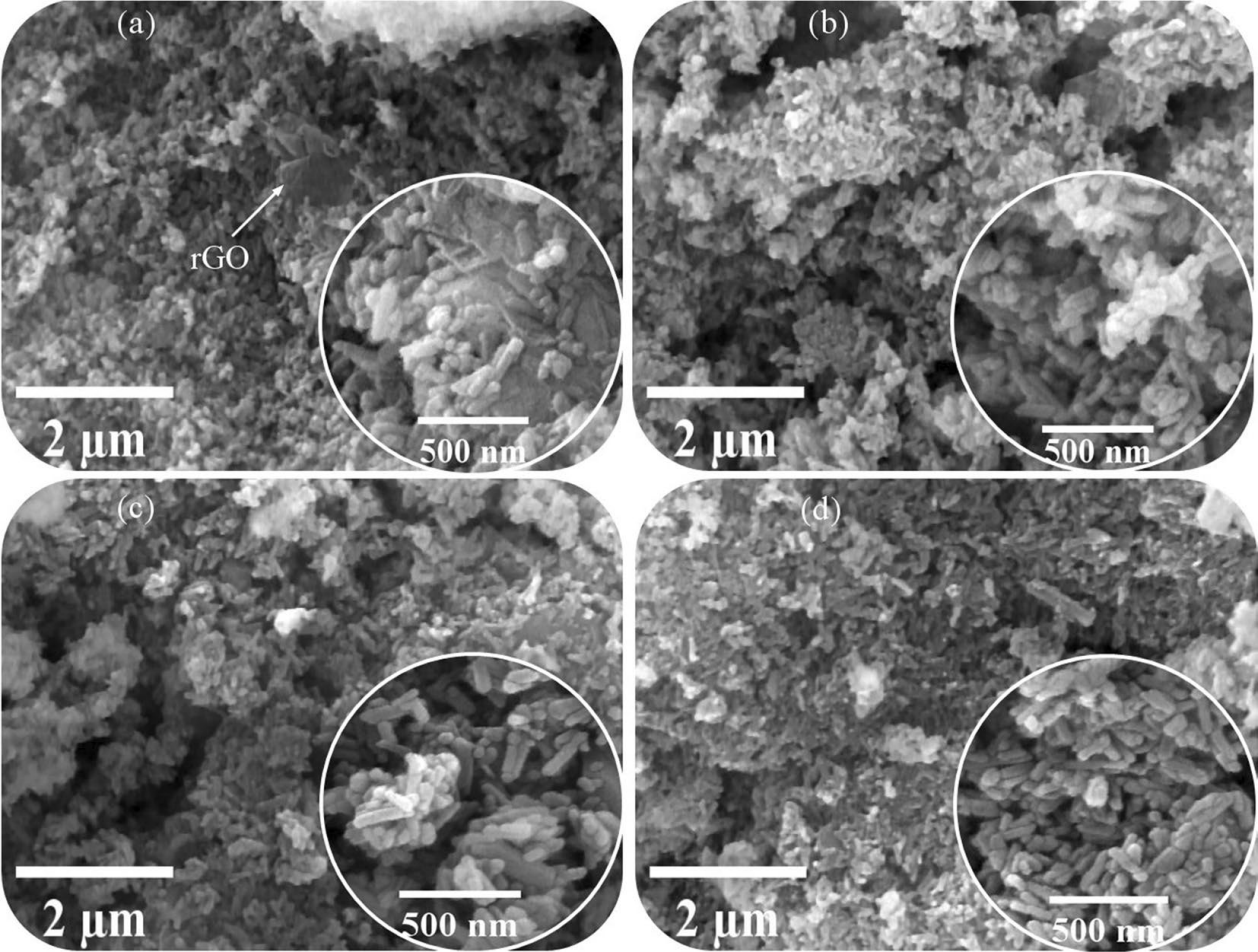
FESEM images of the synthesized powders using **a** volume change (I), **b** argon (II), **c** hydrogen (III), and (d) nitrogen (IV) gas injection (10 bar)

Figure 3 shows the FESEM images of the synthesized powders. These images also confirm the presence of two phases of rGO and HA. Due to the variability of the particle size in these powders, the only issue to be determined from this analysis is the nanorod morphology of the HA particles (in all powders, agglomeration occurs). Therefore, it is better to comment on TEM images [63, 64].

**Fig. 4:**
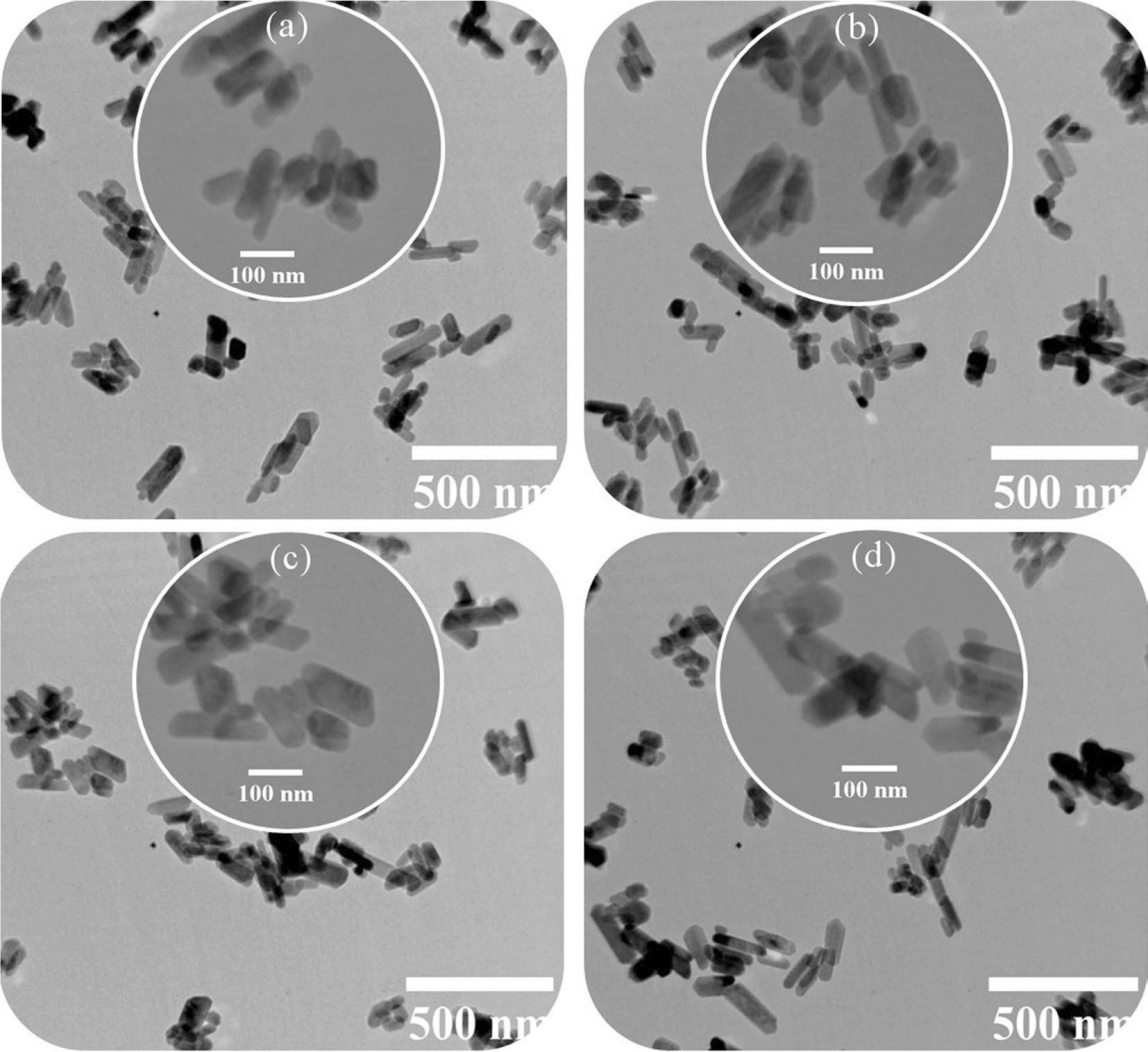
TEM images of the synthesized HA powders using **a** volume change (I), **b** argon (II), **c** hydrogen (III), and **d** nitrogen (IV) gas injection (10 bar)

**Fig. 5:**
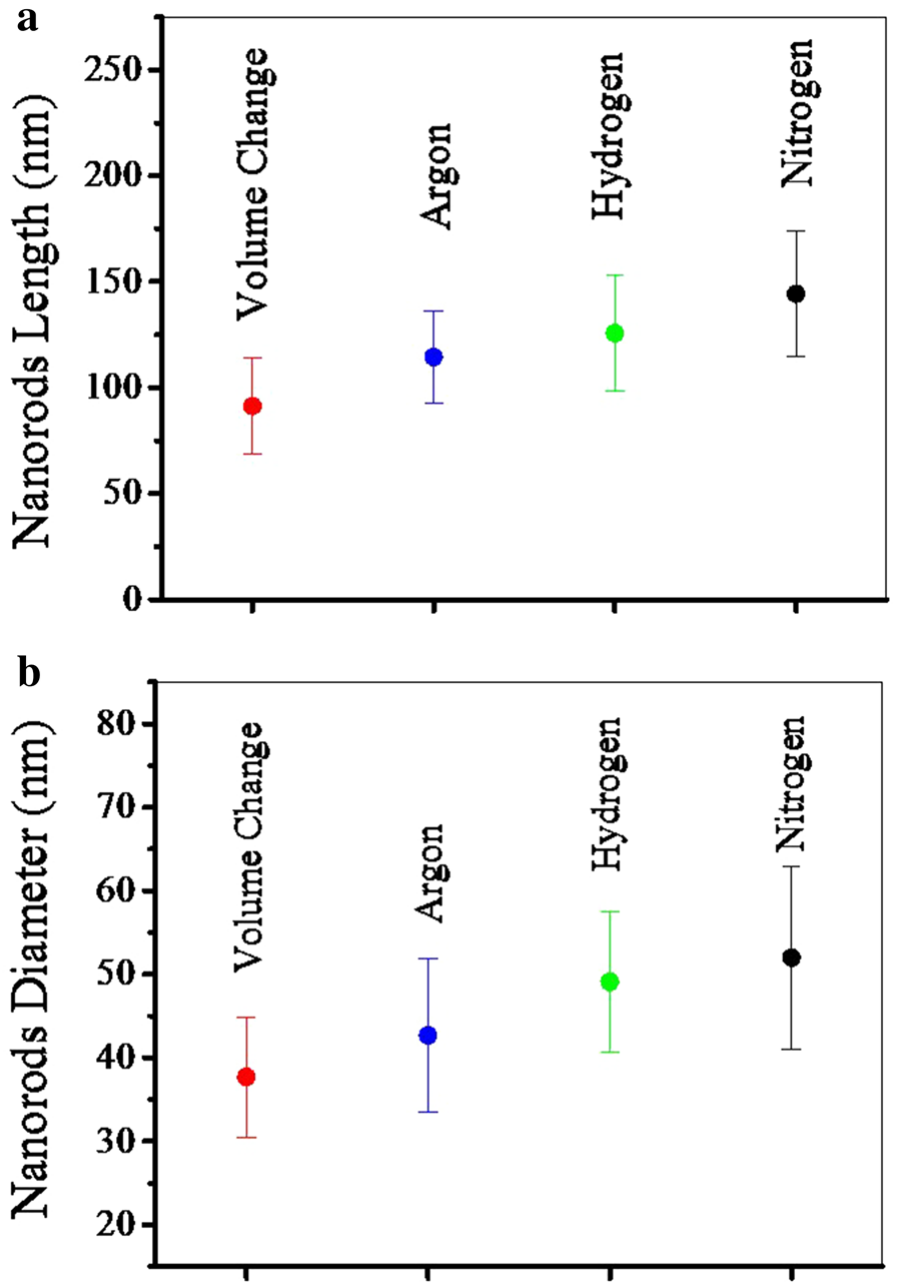
**a** The length size and **b** diameter of the nanorods obtained by images evaluating

Figure 4 shows the TEM images of the synthesized powders. Figure 5 shows the length size and diameter of the nanorods obtained by image analysis. According to the figures, the nitrogen gas has made the particle size somewhat larger. This is due to increased pressure and increased reaction kinetics. Comparison of the two argon and hydrogen gases shows that the hydrogen gas had a greater effect on particle growth. Argon and hydrogen gas partially dissolve in water. This has led to pressure drop at higher temperatures [52, 63].

**Fig. 6:**
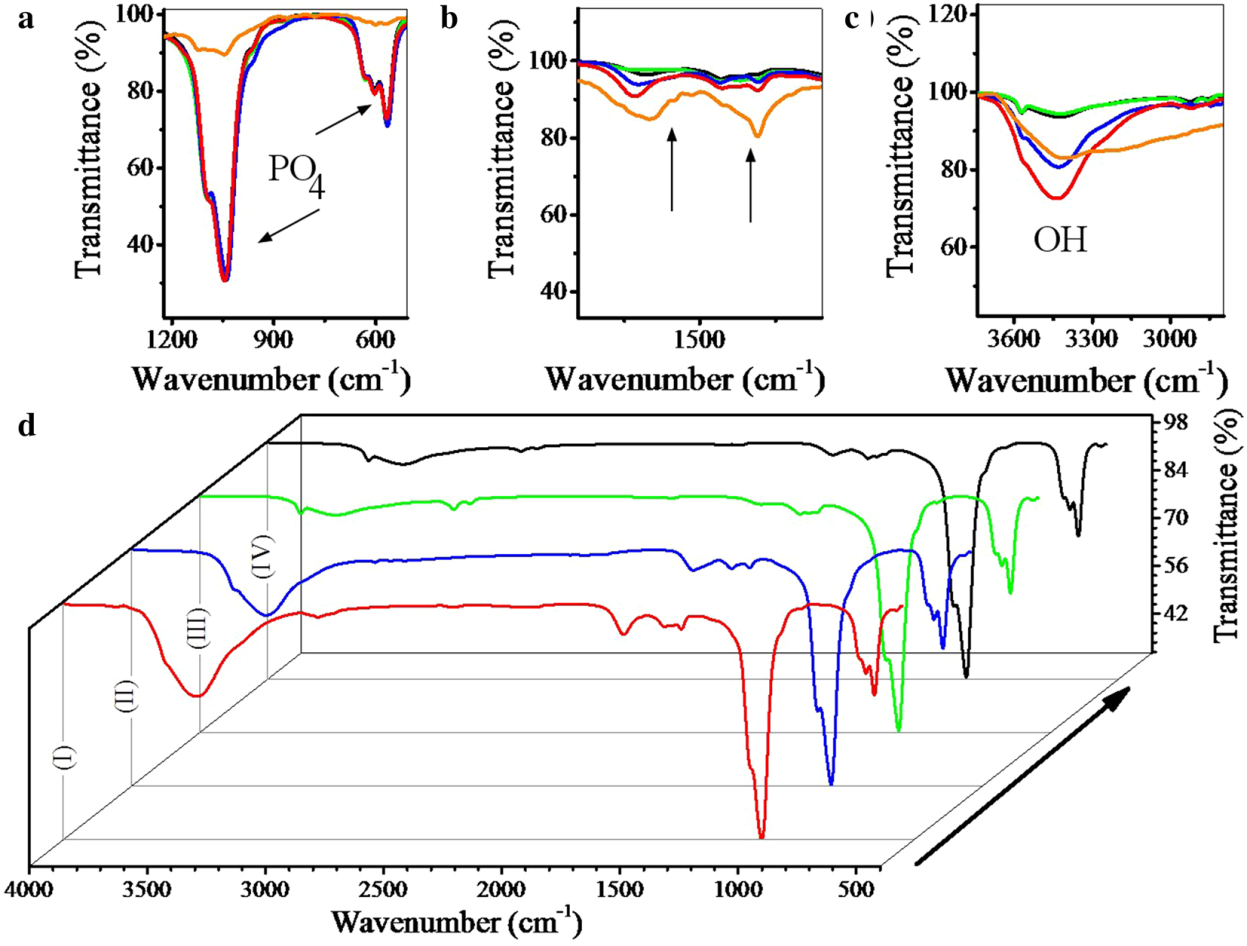
FTIR analysis for the synthesized powders using volume change (I), argon (II), hydrogen (III), nitrogen (IV) gas injection (10 bar), and GO (orange curve)

Figure 6 shows the FTIR analysis for the synthesized powders, and GO (orange curve). Depending on the Fig. 6a, the identified peaks are related to the formation of HA, which is identical in all samples. The peaks shown in Fig. 6b are related to the oxide agents on the graphene oxide surface. It is obvious that after the reduction of GO, the intensity of these peaks decreased and in the sample using nitrogen gas, these peaks almost disappeared. The OH peak is also less intense in the sample synthesized by nitrogen gas injection (Fig. 6c). Given the Fig. 6d and similarity of the peaks, this analysis cannot be further commented on and requires a stronger analysis such as Raman spectroscopy. But before that, it is necessary to discuss the ratio of calcium to phosphate obtained [52, 63, 69].

**Fig. 7:**
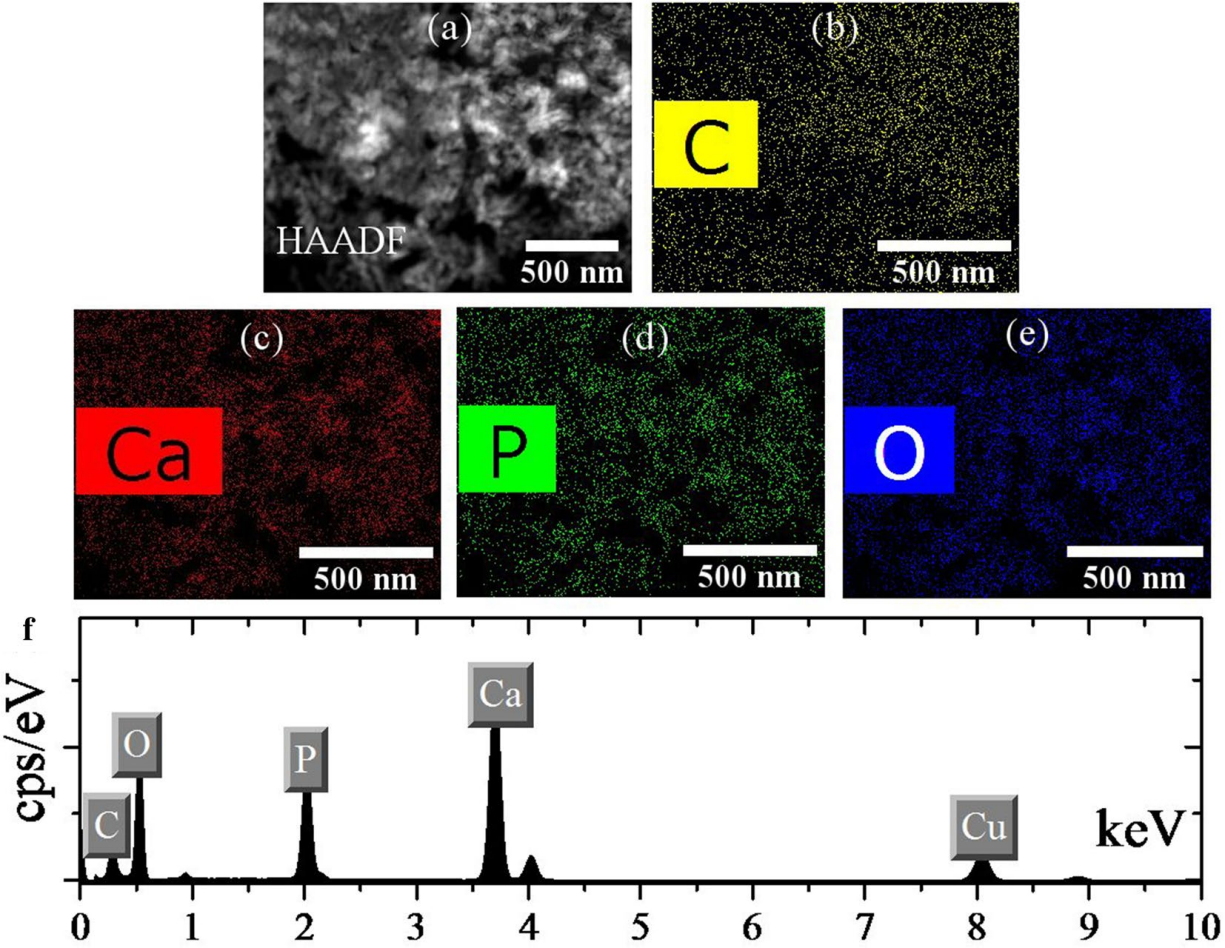
**a** HAADF, **b**–**e** elemental Map, and **f** EDS analysis of the powders synthesized using nitrogen (IV) gas injection (10 bar)

Figure 7 shows the HAADF image, elemental Map, and EDS analysis of IV powders. The residual solution after the hydrothermal process was tested by ICP. The results showed that a very small amount of trace elements remained in solution, resulting in calcium to phosphate ratio of 1.67 in the final powder. Due to the homogeneity of the elemental maps and the results of the EDS analysis, and the ICP analysis output, the synthesized powders are of good quality. Hydrogen and argon gases had previously been evaluated, and here the nitrogen gas was evaluated. The overall result is that the presence of these gases has no negative effect on the quality and purity of the HA powders [65, 69].

**Fig. 8:**
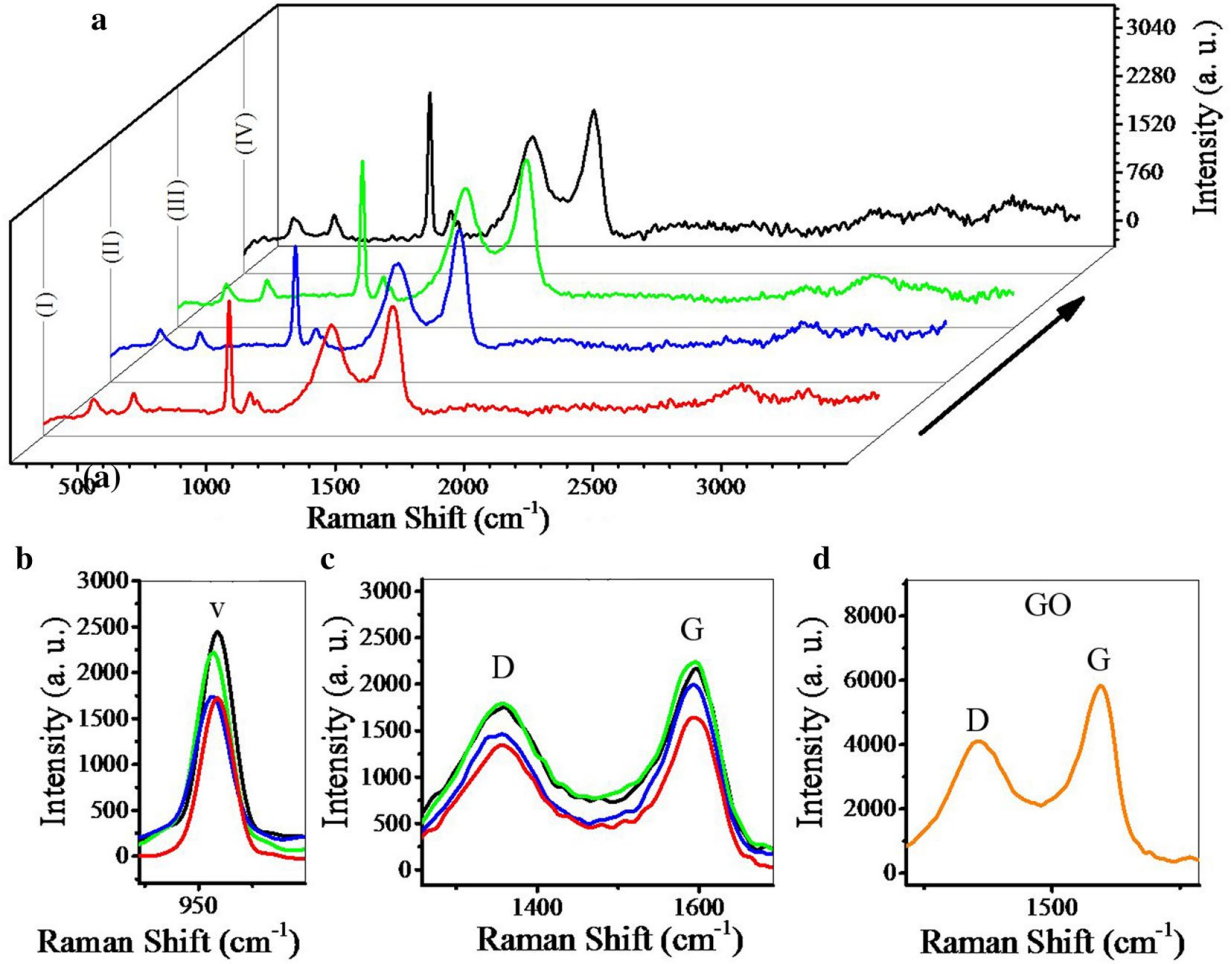
Raman spectroscopy for **a**–**c** synthesized powders using volume change (I), argon (II), hydrogen (III), nitrogen (IV) gas injection (10 bar), and **d** GO

Figure 8 shows the Raman spectroscopy for synthesized powders and GO. Raman spectroscopy is one of the most appropriate methods for evaluating carbon materials. As illustrated in Fig. 8a, all the synthesized powders have similar peaks. The peak located in Raman shift of 960 cm^−1^ is related to the HA structure (Fig. 8b). According to these results, the injection of nitrogen gas increased the intensity of this peak, which is consistent with the results of the XRD analysis. The peaks D and G are related to the graphene structure (Fig. 8c, d). The presence of graphene sheets in all powders has been confirmed so far. To determine the degree of reduction, the ratio of D peak intensity to G peak intensity is considered (ID/IG). In all powders this ratio is increased compared to GO. As a result, GO sheets in all powders are reduced. But this ratio is higher for hydrogen and nitrogen gases than argon, which is indicative of the greater effect of these gases on reduction of GO. Therefore, the mechanical properties of samples synthesized in the presence of nitrogen and hydrogen gases are expected to be greater [63, 65, 69].

**Fig. 9:**
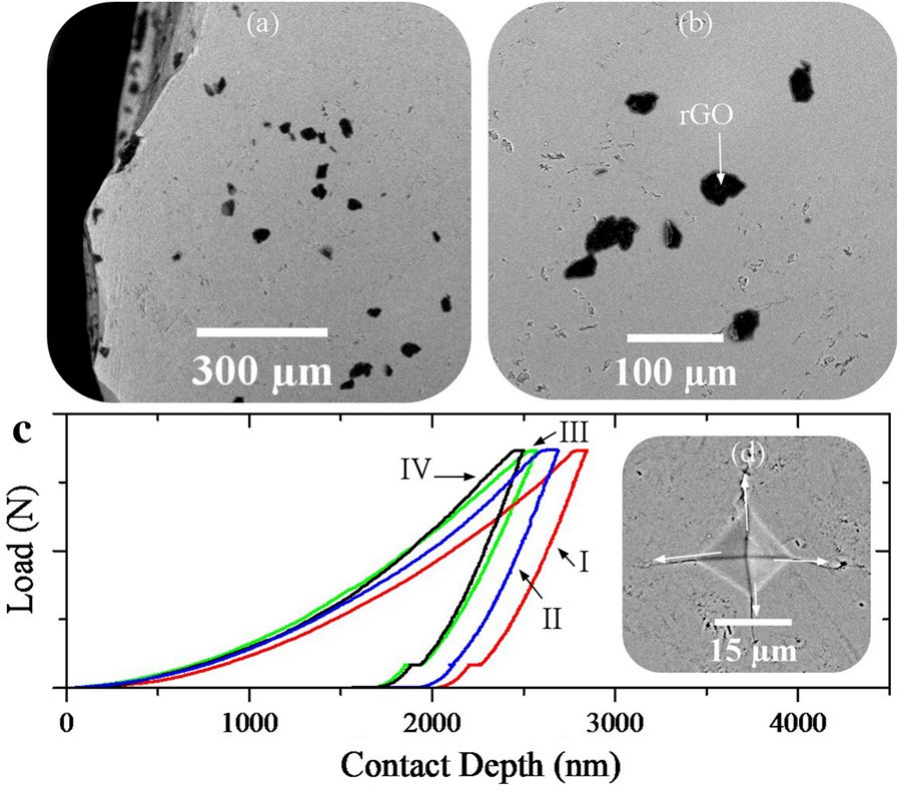
**a**, **b** FESEM images of the sintered powders synthesized using nitrogen (IV) gas injection (10 bar) and **c** load–displacement diagrams for the sintered powders synthesized using volume change (I), argon (II), hydrogen (III), nitrogen (IV) gas injection (10 bar), **d** SEM image of the Vickers affected zone

Figure 9 shows the FESEM images of sintered IV fracture surface and load–displacement diagrams for the sintered samples. The black spots on the images (Fig. 9a, b) are related to graphene sheets decorated with HA nanorods. As can be seen, these graphene sheets play a reinforcing role in three dimensions. Despite the use of the ball milling process, the graphene sheets have not yet been uniformly distributed in the nanocomposite. One reason for this heterogeneity is the size of graphene sheets that are not the same. The second reason is the agglomeration of the HA particles which do not allow the graphene sheets to distribute homogeneously. As illustrated in Fig. 9c, the presence of injected gases decreases the contact depth and shifts the graphs to the left. This transfer is greater for nitrogen gas than others. With this transfer, the slope of the elastic zone also increases, resulting in an increase in the elastic modulus. As the contact depth decreases, the area of the Vickers indenter affected zone will decrease and the hardness will increase [52, 64, 65].

**Fig. 10:**
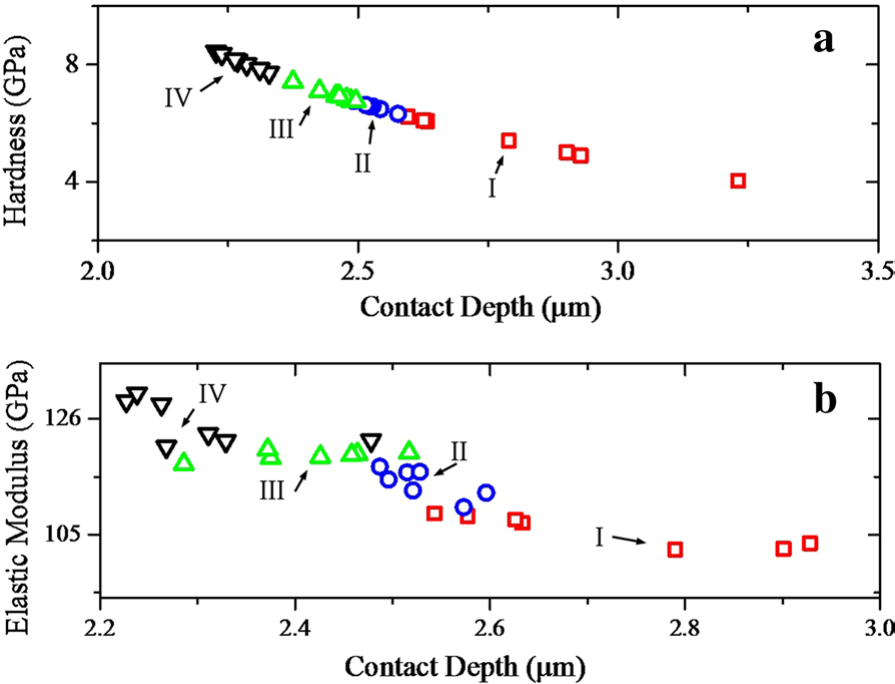
**a** Hardness and **b** elastic modulus of the sintered powders synthesized using volume change (I), argon (II), hydrogen (III), nitrogen (IV) gas injection (10 bar)

Figure 10 shows the diagrams of hardness and elastic modulus variations with the contact depth for the sintered samples. Injection of gases not only increased these properties, but also made these properties more uniform. Nitrogen gas has increased these properties more than other gases (Table 3). The cause of this increase must be considered from two points of view. First, the nitrogen gas is less soluble in water, resulting in increased pressure and increased mechanical properties of HA phase. Second, hydrogen and nitrogen gases react with the surface agents of graphene oxide and cause them to reduce. The effect of nitrogen on the reduction of oxide agents is slightly greater than that of hydrogen. Taken together, these two mechanisms increase the mechanical properties of nanocomposites. Another point seen in the graphs is the increase in hardness with decreasing contact depth. In the case of the elastic modulus, this increase is not uniform. The reason for this non-uniformity is the directional properties of HA [64, 65]. Compared to other hydroxyapatite-based biomaterials, which are currently commercially available, the mechanical properties of the nanocomposites obtained in this study are higher and also the use of nitrogen gas has further increased the mechanical properties and expanded the range of applications of these biomaterials [70–72].

**Table 3:** Mechanical characteristics of the sintered samples

Samples	Hardness (GPa)	Elastic modulus (GPa)
I	5.39 ± 0.75	105.74 ± 2.68
II	6.57 ± 0.12	114.36 ± 2.42
III	6.96 ± 0.21	119.31 ± 0.77
IV	8.10 ± 0.24	125.23 ± 3.77

## Conclusions

The findings of this study indicate that injection of argon, hydrogen and nitrogen gases improved the mechanical properties of rGO-HA nanocomposites. Injection of gases increased crystallinity and particle size of HA, and this increase was greater for nitrogen gas than for others. Injection of these gases increased the rate of GO reduction and in this case the effect of nitrogen gas was greater than the others. The powders synthesized in this study included graphene sheets decorated with HA nanorods. The addition of graphene sheets made the mechanical properties of HA more uniform. Powder characterizations showed that injection of gases increased the HA-related peak intensities in XRD analysis, decreased the GO-peak intensities in FTIR analysis, and increased the ID/IG ratio in Raman spectroscopy. The results of this study could be useful for gas injection applications in hydrothermal autoclave.

## Data Availability

All data provided in the manuscript.

## Abbreviations

GO: Graphene oxide
rGO: Reduced graphene oxide
HA: Hydroxyapatite
TeCP: Tetracalcium phosphate
α-TCP: α- Tricalcium phosphate
β-TCP: β- Tricalcium phosphate
DCPD: Dicalcium phosphate dehydrate
DCPA: Dicalcium phosphate anhydrous
OCP: Octacalcium phosphate
SPS: Spark plasma sintering
HP: Hot pressing
XRD: X-ray diffraction
FTIR: Fourier transform infrared spectroscopy
FESEM: Field emission scanning electron microscopy
TEM: Transmission electron microscopy
HAADF: High angle annular dark field
EDS: Energy-dispersive X-ray spectroscopy
